# Personal

**Published:** 1915-10

**Authors:** 


### PERSONAL.

---

**Announcement of removal, travel, and other matters of interest are requested. Please report errors in the listing of any physician in the State and other directories, that we may co-operate with the State Society in securing a correct list.**

---

Doctors Leet and Selleck (U. of B., 1915), have taken interne services at the Arnot-Ogden Hospital at Elmira.

---

A complimentary dinner was tendered to Dr. R. P. Bush at Cold Brook Club on August 24th, 1915, by the physicians of Chemung County.

---

Dr. Walter J. M. Wurtz of Buffalo, announces the removal of his office from 316 Pearl Street to 1195 Main Street.

---

Dr. Bergren F. Illston of Jamestown is candidate for coroner of Chautauqua Co. on the Republican ticket.

---

Dr. James Wright Putnam of Buffalo returned early in September from a month at Prout's Neck.

---

Dr. W. H. Marcy of Buffalo, returned September 10 from his summer home at Webster, Mass.

Dr. John H. Finerty of Buffalo has bought 96 acres of land with 1300 feet front on Lake Erie in the town of Evans, including a stone house built in 1838 and used as a tavern in stage-coach days, by Levi S. Morsman.

---

Dr. Irving W. Potter, of 420 Franklin Street, Buffalo, announces that after September 1, he will specialize in obstetrics and obstetric surgery.

---

Dr. Julius H. Potter of Buffalo is making a motor tour of a month to the Lake Champlain region, expecting to return early in October.

---

Dr. Karl F. Eschelmann of Buffalo made a motor trip to the east of the state, in September.

---

Dr. Wm. Ward Plummer of Buffalo, returned September 16 from a motor trip to Provincetown, Mass.

---

Dr. Adele Gleason, formerly of Buffalo, is serving in the American Hospital at Neuilly, Paris.

---

Dr. Francis M. O'Gorman of Buffalo has been appointed Attending Gynaecologist to the Sisters' Hospital.

---

Dr. Joseph P. Brennan of Buffalo, announces that he is paying special attention to weak and flat foot, with offices at 399 Delaware Avenue.

---

Dr. George Thomas Moseley of Buffalo was married to Miss Isabella Wells of Concord, Mass., July 6.

---

Dr. Wm .G. Bissell, Bacteriologist of the Buffalo Health Dept., appointed to fill a temporary vacancy in the board of examiners for the state, has received the formal appointment for a term of four years.

---

Dr. Rudolf C. Miller has returned to Buffalo and located at 206 Bissell Avenue.

---

Dr. Homer J. Grant of Buffalo spent August on the New England coast.

---

The home of Dr. M. N. Brooks of Springville was struck by lightning on the night of August 24.

